# Socioeconomic deprivation and ethnicity inequalities in disruption to NHS hospital admissions during the COVID-19 pandemic: a national observational study

**DOI:** 10.1136/bmjqs-2021-013942

**Published:** 2021-11-25

**Authors:** Max Warner, Samantha Burn, George Stoye, Paul P Aylin, Alex Bottle, Carol Propper

**Affiliations:** 1 Institute for Fiscal Studies, London, UK; 2 Centre for Health Economics and Policy Innovation, Imperial College Business School, London, UK; 3 Interfaculty Initiative in Health Policy, Harvard University, Cambridge, Massachusetts, USA; 4 Department of Primary Care and Public Health, Imperial College, London, UK

**Keywords:** COVID-19, health policy, health services research

## Abstract

**Introduction:**

Hospital admissions in many countries fell dramatically at the onset of the COVID-19 pandemic. Less is known about how care patterns differed by patient groups. We sought to determine whether areas with higher levels of socioeconomic deprivation or larger ethnic minority populations saw larger falls in emergency and planned admissions in England.

**Methods:**

We conducted a national observational study of hospital care in the English National Health Service (NHS) in 2019–2020. Weekly volumes of elective (planned) and emergency admissions in 2020 compared with 2019 were calculated for each census area. Multiple linear regression analysis was used to estimate the reductions in volumes for areas in different quintiles of socioeconomic deprivation and ethnic minority populations after controlling for national time trends and local area composition.

**Results:**

Between March and December 2020, there were 35.5% (3.0 million) fewer elective admissions and 22.0% (1.2 million) fewer emergency admissions with a non-COVID-19 primary diagnosis than in 2019. Areas with the largest share of ethnic minority populations experienced a 36.7% (95% CI 24.1% to 49.3%) larger reduction in non-primary COVID-19 emergency admissions compared with those with the smallest. The most deprived areas experienced a 10.1% (95% CI 2.6% to 17.7%) smaller reduction in non-COVID-19 emergency admissions compared with the least deprived. These patterns are not explained by differential prevalence of COVID-19 cases by area.

**Conclusions:**

Even in a healthcare system founded on the principle of equal access for equal need, the impact of COVID-19 on NHS hospital care for non-COVID patients has not been spread evenly by ethnicity and deprivation in England. While we cannot conclusively determine the mechanisms behind these differences, they risk exacerbating prepandemic health inequalities.

## Introduction

Hospital systems around the world have seen large falls in hospital admissions since the beginning of the COVID-19 pandemic,^1–3^ both due to fewer patients seeking care and cancellation of hospital activity,^4^ leading to concerns about worsening health for patients who did not receive care.^5 6^ In many countries, including the UK and USA, deaths and hospitalisations from COVID-19 have been disproportionately concentrated among low-income people and those from minoritised racial and ethnic groups, compounding pre-existing health disparities.^7 8^ Decreases in hospital admissions for non-COVID patients along the same dimensions could further exacerbate these inequalities.

Previous research has shown that the number of hospital admissions during the COVID-19 pandemic fell more for people in more deprived areas^9 10^ and that there were differences in the reductions in primary and hospital care by ethnicity, with emergency hospital admissions decreasing more for black and Asian people than white people.^11 12^ Evidence from the USA has shown relatively small differences in decreases in hospital admissions by insurance status and income and minority composition of local area.^1 4^

The aim of this study is to determine whether areas with higher levels of socioeconomic deprivation or larger ethnic minority populations were more affected by reductions in hospital care during the COVID-19 pandemic in England, after controlling for area demographics and healthcare need.

## Methods

We conducted a national observational study of hospital admissions in the English National Health Service (NHS) during the COVID-19 pandemic. We first present changes in national admissions from March to December 2020 compared with the same period in 2019. We then examine the extent to which different socioeconomic and ethnicity groups experienced different reductions in admissions. As these groups have different medical needs and have experienced different levels of COVID-19 infections, we also examine how admissions changed after controlling for these factors.

### Study setting

The NHS is governed by the principle that access to healthcare should be based solely on clinical need and not ability to pay. International comparisons typically rank its performance highly on ensuring equitable access to healthcare across income groups,^13^ and there is relatively little evidence of socioeconomic inequalities in the use of inpatient care even after adjusting for observed medical needs.^14^ However, deprived areas in England have seen greater numbers of patients hospitalised with COVID-19 than more affluent areas.^15^ Ethnic minority groups have also experienced much greater rates of death and serious illness with COVID-19 than white individuals.^16^

On 17 March 2020, NHS hospitals were instructed to postpone all non-urgent treatments for at least 3 months from mid-April but with ‘full local discretion to wind down elective activity’ in the month prior to this.^17^ Hospitals were also expected to continue as normal with emergency admissions, cancer treatment and other clinically urgent care. England had national lockdowns from 23 March to mid-June 2020 and from 5 November to 2 December 2020 and targeted local restrictions in June–December 2020.

### Data

Our analysis covers all publicly funded emergency and preplanned (elective) admissions for the 56 million residents of England from the beginning of 2019 to the end of 2020. Our source of data is the Hospital Episode Statistics. This administrative database includes deidentified information on all publicly funded hospital care. Each record contains admission and discharge dates, up to 20 diagnoses, admission method, a hospital identifier and patient characteristics including age, sex, ethnicity and the local area of residence. The data do not include privately funded care.

### Sample and variables

#### Outcomes

We extracted all elective and emergency admission episodes between the weeks beginning 7 January 2019 and 21 December 2020. Admissions were classed as elective if patients were admitted from the waiting list or admitted as booked or planned. Admissions were classed as emergency if they were admitted from an accident and emergency (A&E), general practitioner (GP), bed bureau, consultant clinic or A&E of another provider; a baby was born at home as intended and then admitted; or other emergency. All maternity (delivery and birth) admissions were excluded.

We excluded admissions to non-acute NHS hospitals (53 000), private patients treated in NHS hospitals (175 000) and all patients without a valid residence (342 000) or those who do not live in England (126 000).

Local area of residence for each patient is recorded at the middle layer super output area (MSOA) level, a census unit with an average population of 8300 in 2019. All analyses were conducted at the MSOA level. We defined a patient as primary COVID-19 if COVID-19 was recorded as their primary diagnosis on admission. We summed non-primary COVID-19 emergency and elective admissions for each MSOA in each week between January 2019 and December 2020 and calculated weekly admissions in 2020 as a percentage of the closest week in 2019. For example, if admissions in a week in 2020 were 20% lower than in the same week in 2019, the value would be 0.8. We excluded the small number of MSOA week observations with zero admissions in 2019 (four for elective admissions and nine for emergency). This yielded an analysis sample of 346 337 observations at the MSOA week level for elective admissions and 346 332 observations for emergency admissions.

#### Measures of ethnicity and socioeconomic deprivation

To measure socioeconomic deprivation, we assigned each MSOA an index of multiple deprivation (IMD) in 2019. The IMD is the government’s official measure of the population’s deprivation based on seven domains: income, employment, education, health, crime, barriers to housing and services, and living environment.^18^ Deprivation is calculated at the lower layer super output area (approximately a fifth of the size of an MSOA) so we computed a 2019 population weighted average for each MSOA. We categorised MSOAs into five quintiles weighted by 2019 population based on their socioeconomic deprivation. Online supplemental table A1 shows the distribution of the deprivation measure in each quintile.

10.1136/bmjqs-2021-013942.supp1 Supplementary data

To classify ethnic minority status at the MSOA level, we divided MSOAs into five quintiles weighted by 2019 population based on the proportion of admissions in 2019 with a recorded ethnicity that were for non-white patients. We excluded admissions for patients with unknown ethnicity from both the numerator and the denominator (12.6% in 2019). Online supplemental table A2 shows the distribution of the proportion of non-white admissions in 2019 for each quintile. Although there are concerns about the accuracy of HES ethnicity codes^19^ and certain ethnic groups may be over-represented in hospital activity, we used admissions because they are more up-to-date than census population counts at the MSOA level. In online supplemental table A6, we instead used ethnicity data from the 2011 census.

To measure local COVID-19 cases, we calculated the percentage of residents of the MSOA who were admitted to hospital with a primary diagnosis of COVID-19. In online supplemental table A8, we tested using other measures of local COVID-19 cases, including local authority COVID-19 admissions and COVID-19 admissions in the local hospital.

#### Measures of need

We constructed three pre-COVID-19 measures of need at the MSOA level. Mean age and mean percent female were calculated using Office of National Statistics (ONS) 2019 population data.^20^ These capture demographic differences in local area populations that will influence the amount and type of hospital care needed. We also calculated the mean Charlson score of the MSOA residents admitted to hospital in 2019.^21^ The Charlson score measures the number and severity of comorbidities and is a proxy for the underlying health of the local area’s population.

### Statistical analysis

#### Aggregate trends in hospital admissions

We show graphically the changes in national weekly admissions for elective, COVID-19 and (non-primary COVID-19) emergency care relative to the closest week in 2019.

#### Demographics and changes in admissions by socioeconomic and ethnicity groups

We examine changes in the volume of elective and emergency admissions in 2020 compared with 2019 by socioeconomic deprivation and ethnic minority quintiles. These are presented both as percentage changes and as absolute changes per 1000 population. We show how measures of need vary by socioeconomic deprivation and ethnicity quintiles.

#### Adjusted analysis to control for prior usage and need

We use multiple linear regression to identify the effects of socioeconomic deprivation and ethnic minority population on decreases in emergency and elective inpatient admissions, controlling for differences in prior use and need. Regressions are estimated using Ordinary Least Squares (OLS) with standard errors clustered at the MSOA level.^22^

We regressed weekly 2020 admissions at the MSOA level as a percentage of the admissions in the closest week in 2019 on the interactions between indicators for socioeconomic deprivation and ethnic minority population quintiles and a pandemic period indicator. The pandemic period indicator takes the value of 1 from March 2020 onwards and zero otherwise. Examining weekly admissions in 2020 relative to 2019 levels adjusts for prepandemic local hospital use. We included dummies for socioeconomic deprivation and ethnic minority quintiles to control for the different growth rates in volumes between 2019 and 2020 for different quintiles.

We included interactions between the three measures of need and the pandemic period indicator variable to control for changes in hospital admissions during the pandemic due to demographic differences between areas. We included week fixed effects to control for national changes in admissions over time. To examine differences in patterns across care types, we conducted our analysis separately for emergency and elective admissions. The online supplemental appendix 1 includes the full specifications of each regression.

The coefficients for the pandemic interactions with socioeconomic deprivation and ethnicity quintile measure the percentage point difference in the change in admissions during the pandemic for each socioeconomic quintile relative to the least deprived quintile and minority quintile (relative to the quintile with the lowest percentage of minority patients in 2019), respectively. We transformed these percentage point differences into percentage differences using the methodology described in the online supplemental appendix 1.

In an extension, we examined the extent to which local rates of COVID-19 account for any relationship between local socioeconomic deprivation, or ethnicity composition, and changes to admissions. To test this, we included MSOA primary COVID-19 emergency admissions in the regressions. We lag this measure by 1 week because demand and supply responses may be based on past COVID-19 admissions, rather than the current level of admissions.

We present several robustness tests in the online supplemental appendix. Online supplemental tables A3 and A4 present results that include only socioeconomic quintiles and not ethnicity quintiles and vice versa. Online supplemental tables A5 and A6 present results using socioeconomic and ethnicity deciles rather than quintiles. Online supplemental table A7 tests the effect of including mean age squared. Online supplemental tables A8 and A9 tests robustness to the 1 week lag in COVID-19 admissions and uses the number of COVID-19 admissions in the same week and longer lags.

Online supplemental table A10 repeats the analysis of emergency admissions split into low-severity primary diagnoses and high-severity primary diagnoses. We classify diagnosis severity in two ways. First, we calculate mortality rates for each primary diagnosis in 2019 and classify a diagnosis as high severity if its mortality rate is greater than the 75th percentile of the overall mortality distribution. Second, for each diagnosis, we calculate average number of admissions on a week day versus a weekend day and classify diagnoses as non-deferrable if admissions are similar on weekdays and weekends.^23^

## Results

### Aggregate trends in hospital admissions

**Figure 1:**
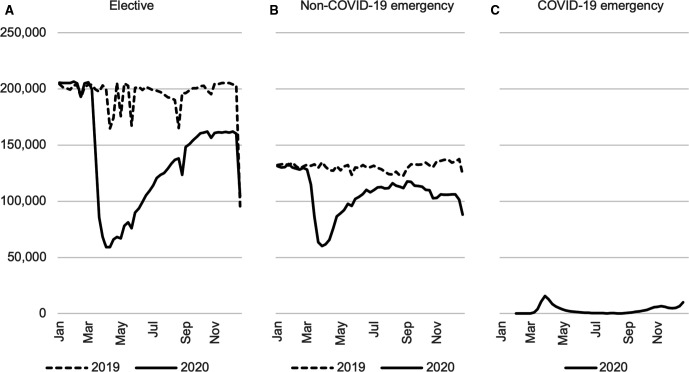
Weekly non-primary COVID-19 elective and emergency admissions 2019 and 2020. Source: authors’ calculations using NHS Digital Hospital Episode Statistics. Note: the week beginning 7 January 2019 is compared with the week beginning 6 January 2020.

Figure 1 shows weekly elective and emergency inpatient admissions in 2019 and 2020 and for comparison primary COVID-19 emergency admissions. There were 17.0 million elective admissions and 12.1 million emergency admissions in our sample. During the pandemic period of March–December 2020, there were 3.0 million fewer elective (35.5%) and 1.2 million (22.0%) fewer emergency admissions than in the same period in 2019.

Elective and emergency admissions were similar in the first 2 months of 2019 and 2020. In March 2020, the number of admissions fell sharply as the number of primary COVID-19 admissions rose. Emergency admissions decreased first, falling in the week beginning 9 March and reached a low of 44.6% of their 2019 level in the final week of March (56.4% when including patients with COVID-19, indicating that the rise in admissions for patients with COVID-19 was nowhere near large enough to offset the overall fall in the total volume of emergency admissions). This rebounded to 93.7% of the 2019 levels by August before falling again to 73.7% in the third week of December. Elective admissions fell from the week beginning 16 March after national guidance was issued to postpone non-urgent care. Elective admissions reached 29.7% of their 2019 level in the first week of April before recovering to 78.9% of their 2019 level in the third week of December.

### Demographics and changes in admissions by socioeconomic and ethnicity groups

Table 1 shows changes in elective and emergency admissions and demographics by socioeconomic deprivation quintiles (panel A) and ethnicity minority status quintiles (panel B). Prior to the pandemic from March to December 2019, the number of elective admissions was almost identical in the least and most socioeconomically deprived areas, but the number of emergency admissions was 38.9% higher in the most deprived areas. Areas with the largest ethnic minority population had 24.1% and 13.5% fewer elective and emergency admissions, respectively.

**Table 1:** Changes in volumes between 2019 and 2020 and local area characteristics by socioeconomic and ethnic minority quintiles

A. Socioeconomic deprivation	Least deprived quintile				Most deprived quintile
Elective admissions					
Elective admissions per 1000 population March–December 2019	145.3	151.3	148.0	143.9	142.7
Elective admissions per 1000 population March–December 2020	93.7	97.6	95.6	93.0	90.2
Percentage change in elective admissions March–December 2020 compared with 2019	−35.5	−35.5	−35.4	−35.4	−36.8
Absolute change in elective admissions per 1000 population March–December 2020 compared with 2019	−51.6	−53.7	−52.4	−51.0	−52.5
Emergency admissions					
Emergency admissions per 1000 population March–December 2019	85.2	91.3	94.7	100.3	118.4
Non-primary COVID-19 emergency admissions per 1000 population March–December 2020	67.4	72.5	74.6	78.2	90.2
Percentage change in non-primary COVID-19 emergency admissions March–December 2020 compared with 2019	−21.0	−20.6	−21.2	−22.1	−23.8
Absolute change in non-primary COVID-19 emergency admissions per 1000 population March–December 2020 compared with 2019	−17.9	−18.8	−20.1	−22.2	−28.1
Total number of primary COVID-19 emergency admissions per 1000 population March–December 2020	2.0	2.2	2.4	3.0	3.7
Area characteristics					
Mean Charlson score 2019 admissions	1.25	1.32	1.33	1.30	1.27
Mean 2019 population age	43.1	42.8	41.4	39.0	36.8
Mean 2019 population % female	51.0	50.9	50.6	50.3	50.4
Mean % ethnic minority 2019 admissions	6.8	7.6	12.2	18.7	20.6

B. Proportion ethnic minority	Lowest % ethnic minority quintile				Highest % ethnic minority quintile
Elective admissions					
Elective admissions per 1000 population March–December 2019	169.8	153.6	144.3	134.6	129.0
Elective admissions per 1000 population March–December 2020	110.0	97.3	92.5	86.2	84.3
Percentage change in elective admissions March–December 2020 compared with 2019	−35.2	−36.7	−35.9	−36.0	−34.7
Absolute change in elective admissions per 1000 population March–December 2020 compared with 2019	−59.8	−56.3	−51.8	−48.4	−44.7
Emergency admissions					
Emergency admissions per 1000 population March–December 2019	103.1	101.7	99.7	96.0	89.3
Non-primary COVID-19 emergency admissions per 1000 population March–December 2020	83.0	81.4	79.2	74.7	64.5
Percentage change in non-primary COVID-19 emergency admissions March–December 2020 compared with 2019	−19.6	−19.9	−20.6	−22.2	−27.7
Absolute change in non-primary COVID-19 emergency admissions per 1000 population March–December 2020 compared with 2019	−20.2	−20.3	−20.5	−21.3	−24.7
Total number of primary COVID-19 emergency admissions per 1000 population March–December 2020	2.2	2.4	2.4	2.8	3.6
Area characteristics					
Mean Charlson score 2019 admissions	1.40	1.31	1.26	1.22	1.27
Mean 2019 population age	44.9	42.7	40.8	38.6	35.2
Mean 2019 population % female	51.2	51.0	50.8	50.5	49.6
Mean socioeconomic deprivation quintile	2.8	2.7	2.7	3.0	3.8

Note: all population levels are for 2019.

Source: authors’ calculations using NHS Digital’s Hospital Episode Statistics and Office for National Statistics (2020).

The last four rows of each panel show demographics and morbidity differed substantially by socioeconomic deprivation and ethnicity quintiles. Areas with the highest socioeconomic deprivation had an average age 6.3 years lower than that of the least deprived, and areas with the highest ethnic minority population had an average age 9.8 years lower than that of areas with the lowest ethnic minority population. There is a less clear pattern for Charlson score, partly because of the age differences. Socioeconomic deprivation and ethnic minority population are positively correlated. The most deprived areas had a much larger percentage of ethnic minority admissions in 2019.

Areas were differentially affected by COVID-19. Areas with the highest socioeconomic deprivation had 84.5% more primary COVID-19 admissions than the least deprived. Areas with the highest share of ethnic minority populations had 64.5% more than areas with the lowest share.

Panel A shows relatively little difference in the percentage falls in elective admissions by socioeconomic deprivation quintile (all falls are around 36%). In absolute terms, there are also relatively small differences across quintiles. For emergency admissions, the most deprived quintile experienced a larger percentage reduction (23.8%) and larger absolute reduction (28.1 per 1000 population) than all other quintiles.

Panel B shows relatively little differences in the percentage falls in elective admissions by ethnicity minority quintile. All falls are around 35%. In absolute terms, the falls are smaller for the highest share quintile areas (44.7 per 1000 population) due to the lower 2019 volumes. For emergency admissions, both the percentage change and the absolute change increased by minority share quintile. The respective percentage falls for the lowest and highest minority quintile were 19.6% and 27.7%. The absolute falls were 20.2 and 24.7 per 1000 population.

### Adjusted analysis to control for prior usage and demographic differences

**Figure 2:**
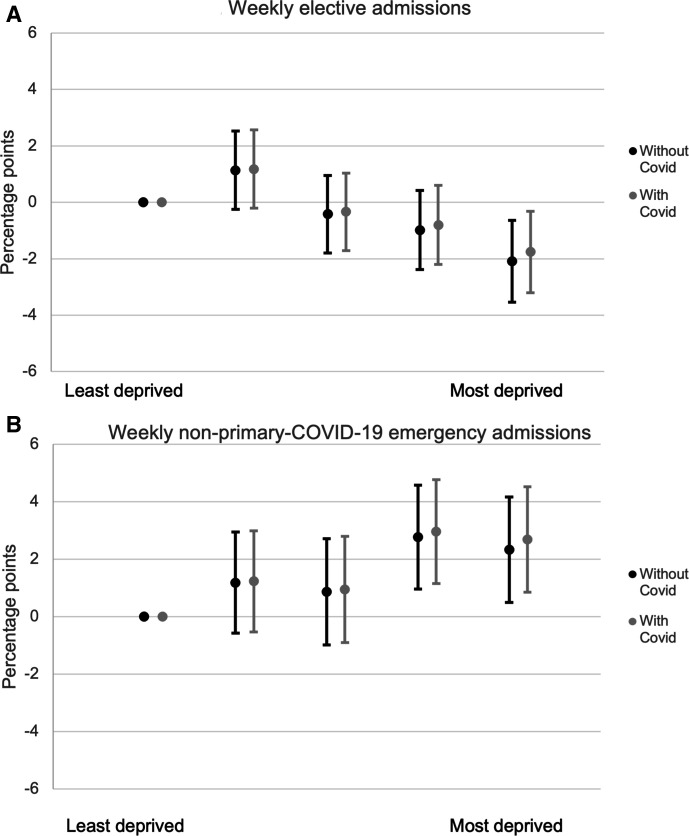
Percentage point change in admissions during COVID-19 period by socioeconomic deprivation quintile relative to the least deprived quintile, controlling for prior need and % ethnic minority quintile.Source: authors’ calculations using NHS Digital Hospital Episode Statistics. Note: panel A shows the coefficients on the interaction between socioeconomic deprivation quintiles and COVID-19 period indicators and the 95% CIs. These measure the difference between each quintile’s mean percentage point reduction in elective admissions during the COVID-19 period relative to the first quintile. The regressions also include week fixed effects, measures of clinical need interacted with the COVID-19 period, and in the second case, local COVID-19 rates lagged by a week. Panel B shows the same for emergency admissions. The online supplemental appendix includes the raw coefficients (specifications 5 and 6).

**Figure 3:**
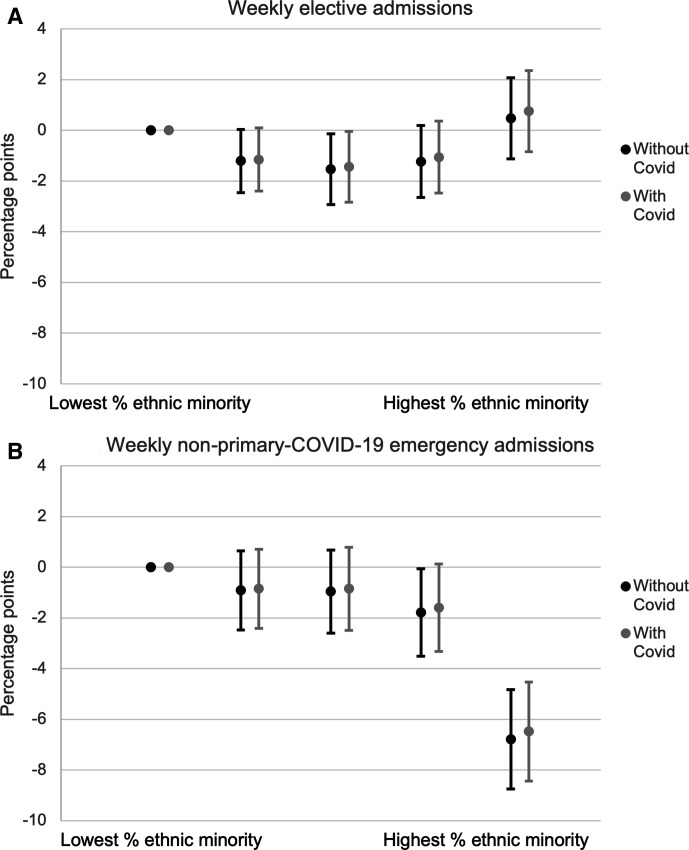
Percentage point change in admissions during COVID-19 period by ethnic minority population quintile, controlling for prior need and socioeconomic deprivation quintile. Source: authors’ calculations using NHS Digital Hospital Episode Statistics. Note: panel A shows the coefficients on the interaction between ethnic minority population quintiles and COVID-19 period indicators and the 95% CIs. These measure the difference between each quintile’s mean percentage point reduction in elective admissions during the COVID-19 period relative to the first quintile. The regressions also include week fixed effects, measures of clinical need interacted with the COVID-19 period and in the second case, local COVID-19 rates lagged by a week. Panel B shows the same for emergency admissions. The online supplemental appendix includes the raw coefficients (specifications 5 and 6).

Figures 2 and 3 show the results from the multivariate regression. Figure 2 plots the coefficients on quintiles of socioeconomic deprivation interacted with the pandemic period indicator. For elective admissions (panel A), there was a 2.1 percentage points (95% CI 0.6 to 3.5) larger fall in elective admissions in the most deprived quintile relative to the fall in the least deprived quintile. This is equivalent to a 5.3% (95% CI 1.5% to 9.0%) larger fall. For emergency care (panel B), more deprived areas experienced smaller reductions in admissions than less deprived areas. There was a 2.3 percentage point (95% CI 0.5 to 4.2) smaller reduction in admissions in the most deprived quintile compared with the least deprived, equivalent to a 10.1% (95% CI 2.6% to 17.7%) smaller fall.

Figure 3 shows the results for ethnic minority percentage quintiles. Panel A shows that areas with the greatest share of non-white patients in 2019 had similar falls in elective admissions in 2020 to those with the smallest share of non-white patients. The greatest fall was in quintile 3, which experienced a 1.5 percentage point (95% CI 0.1 to 2.9) larger fall than the fall in quintile 1, which is equivalent to a 4.1% (95% CI 0.3% to 7.9%) larger reduction. Panel B shows there were large differences across minority status quintiles in the fall in emergency admissions. Areas with the greatest percentage of minority patients in 2019 had an additional fall of 6.8 percentage points (95% CI 4.8 to 8.7) in emergency admissions compared with areas with the smallest percentage of minority patients, equivalent to a 36.7% (95% CI 24.1% to 49.3%) larger fall.

None of the coefficients are changed substantially after additionally controlling for lagged COVID-19 hospital admissions, indicating that differences were not driven by higher COVID-19 prevalence in areas that are more deprived or have larger ethnic minority population. The results are robust to alternate measures of COVID-19 admissions (online supplemental tables A8 and A9) and to use of deciles of socioeconomic deprivation and ethnic minority population (online supplemental tables A5 and A6), inclusion of a quadratic term in age (online supplemental table A7) and use of ethnicity data from the 2011 census instead of 2019 admissions (online supplemental table A6). Online supplemental table A10 repeats the analysis of emergency admissions separately by low-severity and high-severity primary diagnoses and shows a large difference in drops in admission between low and high ethnic minority areas for both types of diagnoses.

## Discussion

### Key results

Inpatient hospital admissions in England fell dramatically at the start of the pandemic in March 2020. We used detailed administrative hospital data that captures the entire public hospital system in England to show that there were 3.0 million fewer elective (35.5%) and 1.2 million (22.0%) fewer emergency admissions in March–December 2020 than in the same period in 2019.

There was a slightly larger fall in elective admissions and a slightly smaller fall in emergency admissions in the most deprived quintile than the least deprived quintile. Areas with higher ethnic minority shares had substantially larger reductions in emergency admissions, but the pattern for elective admissions was less clear.

These differences are approximately equivalent to the most deprived areas losing 3.0 more elective admissions and 2.8 fewer emergency admissions per 1000 residents compared with the least deprived, and areas with the largest ethnic minority populations losing an additional 6.1 emergency admissions per 1000 residents compared with areas with the smallest ethnic minority populations. None of these patterns are explained by the differential prevalence of hospitalised COVID-19 cases in the local area.

### Strengths and limitations

This study benefits from national data with standardised coding. It includes all emergency hospital care and nearly all elective hospital care delivered in England. This provides a nationally representative and large sample that includes all NHS hospitals and covers all areas of England.

The limitations are as follows. First, socioeconomic deprivation and ethnicity are measured at the small area MSOA level rather than at the individual level. As there is variation in socioeconomic status and ethnicity within MSOAs, our estimates will not capture the full extent of differences in changes in hospital use across these groups. Second, the ethnicity data used to classify MSOAs is subject to some error, with 12.6% of patients in 2019 registered as of ‘unknown’ ethnicity. Third, the ethnicity measure is per cent non-white. Within the non-white category are individuals of different ethnicities who may have different access to care, health needs and care-seeking behaviour. Fourth, we do not observe visits that are initiated by the patient. These are visits to emergency departments (as the relevant dataset changed its methodology just before the pandemic) and primary care visits. Nor do we observe referrals for elective care.

A final concern is whether our results are driven by changes to the denominator of our estimates. If populations in different areas changed during the pandemic, because of COVID-19 deaths or pandemic-related population movement, and these changes differed by ethnic minority or socioeconomic deprivation quintile, this could bias our results. While it is not possible to directly account for COVID-19 mortality in our analysis, we can indicate its effect on the magnitude of our estimates. We would expect COVID-19 mortality to affect the elective and emergency results similarly. There were 1.39 deaths with COVID-19 on the death certificate in England during our sample period per 1000 population in 2019.^24^ A percentage of 24.3 of COVID-19 deaths occurred in the fifth most deprived local areas, and 16.6% occurred in the fifth least deprived local areas.^25^ This implies that there were approximately 0.1 more deaths per 1000 in the most deprived quintile relative to the least deprived quintile. Our results imply that the most deprived quintile lost 3.0 additional elective admissions per 1000 and 2.8 fewer emergency admissions per 1000. Even if the difference in mortality between high and low ethnic minority areas is twice as large as for deprivation, at 0.2 deaths per 1 000, this remains far smaller than the difference of 6.1 emergency admissions per 1000 we find between the first and fifth ethnicity quintile.

### Interpretation

These results show that the impacts of COVID-19 on falls in use of hospital care have not been spread evenly across population groups in England. In the case of deprivation, more deprived areas had larger falls in elective admissions and smaller falls in emergency admissions. Areas with high ethnic minority populations had much larger falls in emergency admissions, but there was little difference in elective admissions falls for high and low ethnic minority areas.

We cannot conclusively identify changes in admissions arising from care-seeking behaviour and changes in provider behaviour, but it seems likely that any supply-side response would be in the same direction for elective and emergency admissions and may be larger for elective admissions since these are easier for hospitals to control. This suggests that the larger falls in emergency admissions we see in areas with larger ethnic minority shares may be driven by differences in demand. This is consistent with evidence from the UK and the USA that ethnic minorities have been more likely to avoid seeking care during the pandemic.^26^ In Online supplemental table A10, we split emergency admissions into low-severity and high-severity primary diagnoses. There are larger falls for areas with a high ethnic minority share for both types of diagnoses, supporting our conjecture that the differences by ethnicity were driven by demand-side changes, as it is unlikely that hospitals turned away emergency patients with high-severity diagnoses. The lack of difference in elective admissions falls by ethnic minority share may be driven partly by the composition of admissions across ethnicity. For example, in nephrology, a large elective specialty that saw relatively little COVID-19 disruption, non-white patients made up 30.8% of admissions in 2019 compared with 12.8% across all specialties.

We observe different patterns by deprivation. More deprived areas had larger falls in elective admissions but smaller falls in emergency admissions. The smaller decline in emergency admissions for more deprived areas only occurs in low-severity admissions. This implies that there may be both supply-side and demand-side factors at play.

While we conjecture that the patterns in emergency admissions are driven at least in part by changes in demand-side behaviour, the difference between supply-side and demand-side factors is not clear cut. For example, if patients avoided seeking care due to government messaging, this is a demand-side response to a supply-side intervention. Similarly, if patients attended emergency departments because they were not able to obtain primary care, this is a demand response to supply changes elsewhere in the health system. It is also difficult to distinguish between reductions in need for healthcare, for example, public health measures reducing spread of other communicable diseases, and people being less likely to seek hospital care conditional on need. If the falls in care we observe were driven by individuals avoiding seeking care for medical needs, then the larger declines in emergency care in areas with high ethnic minority share risks exacerbating pre-existing health inequalities.

## Conclusions

In England, there were 35.5% fewer elective admissions and 22.0% fewer non-primary COVID-19 emergency admissions in March–December 2020 than in the same period in 2019. For elective admissions, there was a larger fall in elective admissions for more deprived areas and no difference by ethnic minority population. For emergency admissions, the most deprived areas experienced 10.1% smaller reductions than the least deprived areas, while the areas with the largest ethnic minority populations experienced 36.7% larger reductions than areas with the smallest ethnic minority populations. We conclude that the impacts of COVID-19 on hospital care have not been spread evenly across population groups.

## Data Availability

Data may be obtained from a third party and are not publicly available. The dataset used for this study is an extract from the Hospital Episode Statistics (HES) dataset, held securely at Imperial College London. Users that meet certain requirements can apply to obtain HES data from NHS Digital via the Data Access Request Service (DARS).
