# Supplementary material for: Socioeconomic deprivation and ethnicity inequalities in disruption to NHS hospital admissions during the COVID-19 pandemic: a national observational study
Source: BMJ Qual Saf. 2021 Nov 25:bmjqs-2021-013942. doi: 10.1136/bmjqs-2021-013942 (PMC8627367; doi:10.1136/bmjqs-2021-013942)
Supplement: Supplementary file 1 [file bmjqs-2021-013942supp001.pdf]

Appendix

Quintile definitions

Table A1: Index of Multiple Deprivation (IMD) distribution by socio-economic deprivation quintile

	Minimum	Median	Maximum
1st quintile (least deprived)	2.16	7.86	10.48
2nd quintile	10.48	12.91	15.41
3rd quintile	15.41	18.54	22.04
4th quintile	22.05	26.58	32.09
5th quintile (most deprived)	32.09	40.97	86.86

Table A2: Percentage of 2019 admissions from ethnic minorities distribution by ethnic minority quintile

	Minimum	Median	Maximum
1st quintile (lowest % ethnic minority quintile)	0.000	0.009	0.016
2nd quintile	0.014	0.022	0.033
3rd quintile	0.031	0.050	0.084
4th quintile	0.082	0.134	0.257
5th quintile (highest % ethnic minority quintile)	0.256	0.461	0.953

Note: the maximum of each quintile is larger than the minimum of the next quintile because the percentages in the table are calculated using rounded sums to prevent disclosure rather than the raw sums used to compute the quintiles

Regression specifications

y_{it} is the outcome variable of interest for Middle Layer Super Output Area (MSOA) i in week t. C_t is an indicator for the COVID period. D_i^j is an indicator for socio-economic deprivation quintile j. E_i^j is an indicator for ethnicity quintile j. δ_t is a vector of week fixed effects. X_i^j is MSOA characteristic j. R_{it} is the MSOA COVID rate, lagged by one week.

$$y_{it} = \alpha + \delta_t + \sum_{k=2}^{5} \theta_k^D D_i^k + \sum_{k=2}^{5} \beta_k^D C_t D_i^k + \sum_j \gamma_j C_t X_i^j + \epsilon_{it} \#(1)$$

$$y_{it} = \alpha + \delta_t + \sum_{k=2}^{5} \theta_k^D D_i^k + \sum_{k=2}^{5} \beta_k^D C_t D_i^k + \sum_j \gamma_j C_t X_i^j + \lambda R_{it-1} + \epsilon_{it} \#(2)$$

$$y_{it} = \alpha + \delta_t + \sum_{k=2}^{5} \theta_k^E E_i^k + \sum_{k=2}^{5} \beta_k^E C_t E_i^k + \sum_j \gamma_j C_t X_i^j + \epsilon_{it} \#(3)$$

$$y_{it} = \alpha + \delta_t + \sum_{k=2}^{5} \theta_k^E E_i^k + \sum_{k=2}^{5} \beta_k^E C_t E_i^k + \sum_j \gamma_j C_t X_i^j + \lambda R_{it-1} + \epsilon_{it} \#(4)$$

$$y_{it} = \alpha + \delta_t + \sum_{k=2}^{5} \theta_k^D D_i^k + \sum_{k=2}^{5} \theta_k^E E_i^k + \sum_{k=2}^{5} \beta_k^D C_t D_i^k + \sum_{k=2}^{5} \beta_k^E C_t E_i^k + \sum_j \gamma_j C_t X_i^j + \epsilon_{it} \#(5)$$

$$y_{it} = \alpha + \delta_t + \sum_{k=2}^{5} \theta_k^D D_i^k + \sum_{k=2}^{5} \theta_k^E E_i^k + \sum_{k=2}^{5} \beta_k^D C_t D_i^k + \sum_{k=2}^{5} \beta_k^E C_t E_i^k + \sum_j \gamma_j C_t X_i^j + \lambda R_{it-1} + \epsilon_{it} \#(6)$$

Primary regression results

Table A3: Outcome variable is elective admissions in 2020 normalized by closest week in 2019.

	(1)	(2)	(3)	(4)	(5)	(6)
Intercept	1.078***	1.078***	1.063***	1.063***	1.072***	1.072***
	(0.006)	(0.006)	(0.006)	(0.006)	(0.007)	(0.007)
2nd Income Quintile * Covid	0.014*	0.014**			0.011	0.012*
	(0.007)	(0.007)			(0.007)	(0.007)
3rd Income Quintile * Covid	0.000	0.000			-0.004	-0.003
	(0.007)	(0.007)			(0.007)	(0.007)
4th Income Quintile * Covid	-0.006	-0.004			-0.010	-0.008
	(0.007)	(0.007)			(0.007)	(0.007)
5th Income Quintile * Covid	-0.017**	-0.015**			-0.021***	-0.018**
	(0.007)	(0.007)			(0.007)	(0.007)
2nd Ethnicity Quintile * Covid			-0.009	-0.009	-0.012*	-0.011*
			(0.006)	(0.006)	(0.006)	(0.006)
3rd Ethnicity Quintile * Covid			-0.011	-0.010	-0.015**	-0.014**
			(0.007)	(0.007)	(0.007)	(0.007)
4th Ethnicity Quintile * Covid			-0.006	-0.005	-0.012*	-0.011
			(0.007)	(0.007)	(0.007)	(0.007)
5th Ethnicity Quintile * Covid			0.012	0.014*	0.005	0.008
			(0.008)	(0.008)	(0.008)	(0.008)
Mean Age * Covid	-0.006***	-0.006***	0.003	0.003	-0.005**	-0.005**
	(0.002)	(0.002)	(0.002)	(0.002)	(0.002)	(0.002)
% Female * Covid	0.000	0.000	-0.001	0.000	0.000	0.001
	(0.001)	(0.001)	(0.001)	(0.001)	(0.001)	(0.001)
Mean Charlson * Covid	0.009***	0.009***	0.007***	0.007***	0.008***	0.008***
	(0.001)	(0.001)	(0.001)	(0.001)	(0.001)	(0.001)
Number of Covid Patients		-75.666***		-84.427***		-76.695***
		(5.085)		(5.129)		(5.061)
Num.Obs.	346337	346337	346337	346337	346337	346337
R2	0.415	0.415	0.414	0.415	0.415	0.415

Source: Authors' calculations using NHS Digital's Hospital Episode Statistics.
Note: Standard errors (clustered at the MSOA level) in parentheses. * p < 0.1, ** p < 0.05, *** p < 0.01. Fixed effects are included.

Table A4: Outcome variable is non-primary-COVID-19 emergency admissions in 2020 normalized by closest week in 2019.

	(1)	(2)	(3)	(4)	(5)	(6)
Intercept	1.098***	1.098***	1.078***	1.078***	1.096***	1.096***
	(0.008)	(0.008)	(0.007)	(0.007)	(0.009)	(0.009)
2nd Income Quintile * Covid	0.013	0.013			0.012	0.012
	(0.009)	(0.009)			(0.009)	(0.009)
3rd Income Quintile * Covid	0.009	0.010			0.009	0.009
	(0.009)	(0.009)			(0.009)	(0.009)
4th Income Quintile * Covid	0.029***	0.031***			0.028***	0.030***
	(0.009)	(0.009)			(0.009)	(0.009)
5th Income Quintile * Covid	0.031***	0.035***			0.023**	0.027***
	(0.009)	(0.009)			(0.009)	(0.009)
2nd Ethnicity Quintile * Covid			-0.006	-0.006	-0.009	-0.008
			(0.008)	(0.008)	(0.008)	(0.008)
3rd Ethnicity Quintile * Covid			-0.005	-0.004	-0.010	-0.009
			(0.008)	(0.008)	(0.008)	(0.008)
4th Ethnicity Quintile * Covid			-0.008	-0.007	-0.018**	-0.016*
			(0.009)	(0.009)	(0.009)	(0.009)
5th Ethnicity Quintile * Covid			-0.047***	-0.045***	-0.068***	-0.065***
			(0.010)	(0.010)	(0.010)	(0.010)
Mean Age * Covid	0.024***	0.023***	0.014***	0.014***	0.006**	0.007***
	(0.002)	(0.002)	(0.002)	(0.002)	(0.002)	(0.002)
% Female * Covid	-0.006***	-0.006***	-0.009***	-0.009***	-0.008***	-0.008***
	(0.002)	(0.002)	(0.002)	(0.002)	(0.002)	(0.002)
Mean Charlson * Covid	0.000	0.000	0.001	0.001	0.002	0.002
	(0.002)	(0.002)	(0.002)	(0.002)	(0.002)	(0.002)
Number of Covid Patients		-94.773***		-90.687***		-83.223***
		(6.392)		(6.429)		(6.346)
Num.Obs.	346332	346332	346332	346332	346332	346332
R2	0.108	0.108	0.109	0.109	0.109	0.110

Source: Authors' calculations using NHS Digital's Hospital Episode Statistics.
Note: Standard errors (clustered at the MSOA level) in parentheses. * p < 0.1, ** p < 0.05, *** p < 0.01. Fixed effects are included.

Coefficient transformations

To calculate the percentage point fall for each quintile from specifications (5) and (6) we sum the implied COVID period coefficient, relevant quintile coefficient and an adjustment term to account for the different base group when both sets of quintile indicators are included. This adjustment ensures that the omitted group when interpreting the deprivation quintile coefficients is the lowest deprivation quintile with the average (for the group) percent ethnic minority quintile and vice versa. The implied COVID period coefficient is the difference between the mean week fixed effect during and before the COVID period. For specification (5) this is -0.3805 for elective admissions and -0.2164 for emergency admissions. The adjustment term is based on the joint distribution of the two groups of quintiles. For calculating falls for socio-economic deprivation quintiles, the adjustment term is equal to the average $\frac{340}{1390}(\beta_2^E - \theta_2^E) + \frac{436}{1390}(\beta_3^E - \theta_3^E) + \frac{315}{1390}(\beta_4^E - \theta_4^E) + \frac{52}{1390}(\beta_5^E - \theta_5^E)$ and for ethnicity quintiles, the adjustment term is $\frac{403}{1465}(\beta_2^D - \theta_2^D) + \frac{388}{1465}(\beta_3^D - \theta_3^D) + \frac{234}{1465}(\beta_4^D - \theta_4^D) + \frac{193}{1465}(\beta_5^D - \theta_5^D)$. For specification (5) this is -0.0164 for the socio-economic gradient in elective admissions, -0.0138 for the socio-economic gradient in emergency admissions, 0.0069 for the ethnicity gradient in elective admissions and 0.0312 for the ethnicity gradient in emergency admissions. Confidence intervals on the percentage differences between different percentage points changes are calculated using the delta method.

To calculate the approximate absolute change in elective and emergency admissions in the discussion section, we multiply the coefficients for the fifth quintiles from specification (5) by the number of admissions for the same quintile in March to December 2019. For elective admissions, this was 1,606,000 for the fifth socio-economic quintile and 1,452,000 for fifth ethnicity quintile. For emergency admissions, this was 1,332,000 for the fifth socio-economic quintile and 1,005,000 for the fifth ethnicity quintile. We then divide by the population of the quintile, which was 11,258,170 for the fifth socio-economic quintile and 11,257,630 for the fifth ethnicity quintile.[1]

Robustness tests

Income deprivation and ethnicity deciles

Same specifications as (5) and (6) but D'^j_i and E'^j_i refer to socio-economic and ethnicity deciles respectively.

$$y_{it} = \alpha + \delta_t + \sum_{k=2}^{10} \theta_k^D D_i^k + \sum_{k=2}^{10} \theta_k^E E_i^k + \sum_{k=2}^{10} \beta_k^D C_t D_i^k + \sum_{k=2}^{10} \beta_k^E C_t E_i^k + \sum_j \gamma_j C_t X_i^j + \epsilon_{it} \#(7)$$

$$y_{it} = \alpha + \delta_t + \sum_{k=2}^{10} \theta_k^D D_i^k + \sum_{k=2}^{10} \theta_k^E E_i^k + \sum_{k=2}^{10} \beta_k^D C_t D_i^k + \sum_{k=2}^{10} \beta_k^E C_t E_i^k + \sum_j \gamma_j C_t X_i^j + \lambda R_{it-1} + \epsilon_{it} \#(8)$$

Table A5: Outcome variable is non-primary-COVID-19 admissions in 2020 normalized by closest week in 2019.

	Elective		Emergency	
	(7)	(8)	(7)	(8)
Intercept	1.068***	1.068***	1.105***	1.106***
	(0.010)	(0.010)	(0.013)	(0.013)
2nd Income Decile * Covid	-0.001	-0.001	0.013	0.013
	(0.010)	(0.010)	(0.013)	(0.013)
3rd Income Decile * Covid	0.016	0.016	0.015	0.015
	(0.010)	(0.010)	(0.013)	(0.013)
4th Income Decile * Covid	0.004	0.005	0.021	0.022*
	(0.010)	(0.010)	(0.013)	(0.013)
5th Income Decile * Covid	-0.014	-0.013	0.006	0.007
	(0.010)	(0.010)	(0.014)	(0.014)
6th Income Decile * Covid	0.002	0.003	0.022*	0.023*
	(0.010)	(0.010)	(0.013)	(0.013)
7th Income Decile * Covid	-0.008	-0.007	0.032**	0.034***
	(0.010)	(0.010)	(0.013)	(0.013)
8th Income Decile * Covid	-0.015	-0.013	0.033**	0.035***
	(0.010)	(0.010)	(0.013)	(0.013)
9th Income Decile * Covid	-0.019*	-0.016	0.028**	0.031**
	(0.011)	(0.011)	(0.013)	(0.013)
10th Income Decile * Covid	-0.027***	-0.023**	0.027**	0.031**
	(0.010)	(0.010)	(0.013)	(0.013)
2nd Ethnicity Decile * Covid	-0.027***	-0.026***	0.004	0.005
	(0.009)	(0.009)	(0.011)	(0.011)
3rd Ethnicity Decile * Covid	-0.024***	-0.023***	-0.008	-0.007
	(0.009)	(0.009)	(0.011)	(0.011)
4th Ethnicity Decile * Covid	-0.027***	-0.026***	-0.009	-0.008
	(0.009)	(0.009)	(0.011)	(0.011)
5th Ethnicity Decile * Covid	-0.029***	-0.028***	-0.003	-0.001
	(0.010)	(0.010)	(0.011)	(0.011)
6th Ethnicity Decile * Covid	-0.030***	-0.028***	-0.017	-0.015
	(0.010)	(0.010)	(0.012)	(0.012)
7th Ethnicity Decile * Covid	-0.033***	-0.031***	-0.013	-0.011

	(0.010)	(0.010)	(0.013)	(0.013)
8th Ethnicity Decile * Covid	-0.021**	-0.018*	-0.026**	-0.024**
	(0.010)	(0.010)	(0.012)	(0.012)
9th Ethnicity Decile * Covid	-0.014	-0.011	-0.068***	-0.065***
	(0.011)	(0.011)	(0.014)	(0.014)
10th Ethnicity Decile * Covid	-0.007	-0.003	-0.077***	-0.072***
	(0.011)	(0.011)	(0.012)	(0.012)
Mean Age * Covid	-0.006***	-0.005**	0.003	0.004
	(0.002)	(0.002)	(0.003)	(0.003)
% Female * Covid	0.001	0.001	-0.008***	-0.008***
	(0.001)	(0.001)	(0.002)	(0.002)
Mean Charlson * Covid	0.008***	0.008***	0.003*	0.003*
	(0.001)	(0.001)	(0.002)	(0.002)
Number of Covid Patients		-75.188***		-79.159***
		(5.044)		(6.314)
Num.Obs.	346337	346337	346332	346332
R2	0.415	0.416	0.110	0.110

Source: Authors' calculations using NHS Digital's Hospital Episode Statistics.
Note: Standard errors (clustered at the MSOA level) in parentheses. * p < 0.1, ** p < 0.05, *** p < 0.01. Fixed effects are included.

2011 Census ethnicity quintiles

Specifications are the same as (5) and (6), but using 2011 census data to classify the ethnicity quintiles.

Table A6: Outcome variable is non-primary-COVID-19 admissions in 2020 normalized by closest week in 2019.

	Elective		Emergency	
	(5)	(6)	(5)	(6)
Intercept	1.078***	1.078***	1.104***	1.104***
	(0.007)	(0.007)	(0.009)	(0.009)
2nd Income Quintile * Covid	0.013*	0.014*	0.012	0.013
	(0.007)	(0.007)	(0.009)	(0.009)
3rd Income Quintile * Covid	-0.002	-0.001	0.010	0.011
	(0.007)	(0.007)	(0.009)	(0.009)
4th Income Quintile * Covid	-0.008	-0.006	0.028***	0.030***
	(0.007)	(0.007)	(0.009)	(0.009)
5th Income Quintile * Covid	-0.019**	-0.016**	0.024**	0.028***
	(0.007)	(0.007)	(0.009)	(0.009)
2nd Ethnicity Quintile * Covid	0.001	0.001	0.010	0.011
	(0.006)	(0.006)	(0.008)	(0.008)
3rd Ethnicity Quintile * Covid	-0.001	0.000	0.001	0.002
	(0.007)	(0.007)	(0.008)	(0.008)
4th Ethnicity Quintile * Covid	-0.004	-0.002	-0.013	-0.011
	(0.007)	(0.007)	(0.009)	(0.009)
5th Ethnicity Quintile * Covid	0.011	0.014*	-0.055***	-0.052***
	(0.008)	(0.008)	(0.010)	(0.010)
Mean Age * Covid	-0.004**	-0.004*	0.006**	0.007***
	(0.002)	(0.002)	(0.003)	(0.003)
% Female * Covid	0.000	0.001	-0.009***	-0.009***
	(0.001)	(0.001)	(0.002)	(0.002)
Mean Charlson * Covid	0.009***	0.008***	0.003*	0.003
	(0.001)	(0.001)	(0.002)	(0.002)
Number of Covid Patients		-76.909***		-83.443***
		(5.055)		(6.347)
Num.Obs.	346337	346337	346332	346332
R2	0.415	0.415	0.109	0.109

Source: Authors' calculations using NHS Digital's Hospital Episode Statistics.

Note: Standard errors (clustered at the MSOA level) in parentheses. * p < 0.1, ** p < 0.05, *** p < 0.01. Fixed effects are included.

Additional local need controls

Specifications are the same as (5) and (6), but all include the MSOA's population mean age in 2019 (X_i^1) squared.

$$y_{it} = \alpha + \delta_t + \sum_{k=2}^{5} \theta_k^D D_i^k + \sum_{k=2}^{5} \theta_k^E E_i^k + \sum_{k=2}^{5} \beta_k^D C_t D_i^k + \sum_{k=2}^{5} \beta_k^E C_t E_i^k + \sum_{j} \gamma_j C_t X_i^j + \gamma_1' C_t \left(X_i^1\right)^2 + \epsilon_{it} \#(9)$$

$$y_{it} = \alpha + \delta_t + \sum_{k=2}^{5} \theta_k^D D_i^k + \sum_{k=2}^{5} \theta_k^E E_i^k + \sum_{k=2}^{5} \beta_k^D C_t D_i^k + \sum_{k=2}^{5} \beta_k^E C_t E_i^k + \sum_{j} \gamma_j C_t X_i^j + \gamma_1' C_t \left(X_i^1\right)^2 + \lambda R_{it-1} + \epsilon_{it} \#(10)$$

Table A7: Outcome variable is non-primary-COVID-19 admissions in 2020 normalized by closest week in 2019.

	Elective		Emergency	
	(9)	(10)	(9)	(10)
Intercept	1.072***	1.072***	1.096***	1.096***
	(0.007)	(0.007)	(0.009)	(0.009)
2nd Income Quintile * Covid	0.012	0.012*	0.012	0.013
	(0.007)	(0.007)	(0.009)	(0.009)
3rd Income Quintile * Covid	-0.004	-0.003	0.009	0.010
	(0.007)	(0.007)	(0.009)	(0.009)
4th Income Quintile * Covid	-0.010	-0.008	0.028***	0.030***
	(0.007)	(0.007)	(0.009)	(0.009)
5th Income Quintile * Covid	-0.022***	-0.018**	0.022**	0.026***
	(0.007)	(0.007)	(0.009)	(0.009)
2nd Ethnicity Quintile * Covid	-0.010	-0.010	-0.006	-0.005
	(0.006)	(0.006)	(0.008)	(0.008)
3rd Ethnicity Quintile * Covid	-0.013*	-0.012*	-0.006	-0.005
	(0.007)	(0.007)	(0.008)	(0.008)
4th Ethnicity Quintile * Covid	-0.011	-0.009	-0.015*	-0.013
	(0.007)	(0.007)	(0.009)	(0.009)
5th Ethnicity Quintile * Covid	0.003	0.006	-0.070***	-0.067***
	(0.008)	(0.008)	(0.010)	(0.010)
Mean Age * Covid	-0.006***	-0.005**	0.006**	0.006**
	(0.002)	(0.002)	(0.003)	(0.002)
Mean Age Squared * Covid	0.003***	0.002**	0.005***	0.005***

	(0.001)	(0.001)	(0.001)	(0.001)
% Female * Covid	0.001	0.001	-0.007***	-0.007***
	(0.001)	(0.001)	(0.002)	(0.002)
Mean Charlson * Covid	0.009***	0.009***	0.003*	0.003*
	(0.001)	(0.001)	(0.002)	(0.002)
Number of Covid Patients		-75.632***		-81.234***
		(5.061)		(6.336)
Num.Obs.	346337	346337	346332	346332
R2	0.415	0.415	0.109	0.110

Source: Authors' calculations using NHS Digital's Hospital Episode Statistics.
Note: Standard errors (clustered at the MSOA level) in parentheses. * $p < 0.1$, ** $p < 0.05$, *** $p < 0.01$. Fixed effects are included.

Alternative measures of local COVID rates

Number of COVID patients from the local authority (R'_{it}) and in the local hospital (R''_{it}) are defined analogously to the number of COVID patients in MSOA used in the specification (6). We map MSOAs to local authorities (2019 boundaries) using an ONS mapping (source to population data). We map MSOAs to a unique local hospital defined as the hospital that received the most emergency admissions from the MSOA in 2019.

$$y_{it} = \alpha + \delta_t + \sum_{k=2}^{5} \theta_k^D D_i^k + \sum_{k=2}^{5} \theta_k^E E_i^k + \sum_{k=2}^{5} \beta_k^D C_t D_i^k + \sum_{k=2}^{5} \beta_k^E C_t E_i^k + \sum_j \gamma_j C_t X_i^j + \lambda R_{it} + \epsilon_{it} \#(11)$$

$$y_{it} = \alpha + \delta_t + \sum_{k=2}^{5} \theta_k^D D_i^k + \sum_{k=2}^{5} \theta_k^E E_i^k + \sum_{k=2}^{5} \beta_k^D C_t D_i^k + \sum_{k=2}^{5} \beta_k^E C_t E_i^k + \sum_j \gamma_j C_t X_i^j + \lambda R_{it-2} + \epsilon_{it} \#(12)$$

$$y_{it} = \alpha + \delta_t + \sum_{k=2}^{5} \theta_k^D D_i^k + \sum_{k=2}^{5} \theta_k^E E_i^k + \sum_{k=2}^{5} \beta_k^D C_t D_i^k + \sum_{k=2}^{5} \beta_k^E C_t E_i^k + \sum_j \gamma_j C_t X_i^j + \lambda R_{it-3} + \epsilon_{it} \#(13)$$

$$y_{it} = \alpha + \delta_t + \sum_{k=2}^{5} \theta_k^D D_i^k + \sum_{k=2}^{5} \theta_k^E E_i^k + \sum_{k=2}^{5} \beta_k^D C_t D_i^k + \sum_{k=2}^{5} \beta_k^E C_t E_i^k + \sum_j \gamma_j C_t X_i^j + \lambda R'_{it-1} + \epsilon_{it} \#(14)$$

$$y_{it} = \alpha + \delta_t + \sum_{k=2}^{5} \theta_k^D D_i^k + \sum_{k=2}^{5} \theta_k^E E_i^k + \sum_{k=2}^{5} \beta_k^D C_t D_i^k + \sum_{k=2}^{5} \beta_k^E C_t E_i^k + \sum_j \gamma_j C_t X_i^j + \lambda R''_{it-1} + \epsilon_{it} \#(15)$$

Table A8: Outcome variable is non-primary-COVID-19 elective admissions in 2020 normalized by closest week in 2019.

	(11)	(6)	(12)	(13)	(14)	(15)
Intercept	1.072***	1.072***	1.072***	1.072***	1.072***	1.072***
	(0.007)	(0.007)	(0.007)	(0.007)	(0.007)	(0.007)
2nd Income Quintile * Covid	0.012*	0.012*	0.012*	0.012*	0.012*	0.012*
	(0.007)	(0.007)	(0.007)	(0.007)	(0.007)	(0.007)
3rd Income Quintile * Covid	-0.003	-0.003	-0.003	-0.003	-0.002	-0.003
	(0.007)	(0.007)	(0.007)	(0.007)	(0.007)	(0.007)

4th Income Quintile * Covid	-0.008	-0.008	-0.008	-0.008	-0.006	-0.007
	(0.007)	(0.007)	(0.007)	(0.007)	(0.007)	(0.007)
5th Income Quintile * Covid	-0.018**	-0.018**	-0.018**	-0.018**	-0.013*	-0.014*
	(0.007)	(0.007)	(0.007)	(0.007)	(0.007)	(0.007)
2nd Ethnicity Quintile * Covid	-0.012*	-0.011*	-0.012*	-0.012*	-0.011*	-0.011*
	(0.006)	(0.006)	(0.006)	(0.006)	(0.006)	(0.006)
3rd Ethnicity Quintile * Covid	-0.014**	-0.014**	-0.014**	-0.015**	-0.013*	-0.013*
	(0.007)	(0.007)	(0.007)	(0.007)	(0.007)	(0.007)
4th Ethnicity Quintile * Covid	-0.011	-0.011	-0.011	-0.011	-0.008	-0.008
	(0.007)	(0.007)	(0.007)	(0.007)	(0.007)	(0.007)
5th Ethnicity Quintile * Covid	0.008	0.008	0.007	0.007	0.013	0.013
	(0.008)	(0.008)	(0.008)	(0.008)	(0.008)	(0.008)
Mean Age * Covid	-0.005**	-0.005**	-0.005**	-0.005**	-0.004**	-0.004**
	(0.002)	(0.002)	(0.002)	(0.002)	(0.002)	(0.002)
% Female * Covid	0.001	0.001	0.001	0.000	0.001	0.001
	(0.001)	(0.001)	(0.001)	(0.001)	(0.001)	(0.001)
Mean Charlson * Covid	0.008***	0.008***	0.008***	0.008***	0.008***	0.007***
	(0.001)	(0.001)	(0.001)	(0.001)	(0.001)	(0.001)
Number of Covid Patients from MSOA (Lagged 0 Weeks)	-68.583***					
	(5.726)					
Number of Covid Patients from MSOA (Lagged 1 Week)		-76.695***				
		(5.061)				
Number of Covid Patients from MSOA (Lagged 2 Weeks)			-73.251***			
			(5.097)			
Number of Covid Patients from MSOA (Lagged 3 Weeks)				-72.331***		
				(5.196)		
Number of Covid Patients from Local Authority (Lagged 1 Week)					-328.247***	
					(13.428)	

Number of Covid Patients in Local Hospital (Lagged 1 Week)						-390.287***
						(14.936)
Num.Obs.	346337	346337	346337	346337	346337	346337
R2	0.415	0.415	0.415	0.415	0.417	0.417

Source: Authors' calculations using NHS Digital's Hospital Episode Statistics.
Note: Standard errors (clustered at the MSOA level) in parentheses. * p < 0.1, ** p < 0.05, *** p < 0.01. Fixed effects are included.

Table A9: Outcome variable is non-primary-COVID-19 emergency admissions in 2020 normalized by closest week in 2019.

	(6)	(6)	(6)	(6)	(6)	(6)
Intercept	1.097***	1.096***	1.096***	1.096***	1.097***	1.097***
	(0.009)	(0.009)	(0.009)	(0.009)	(0.009)	(0.009)
2nd Income Quintile * Covid	0.012	0.012	0.012	0.012	0.013	0.012
	(0.009)	(0.009)	(0.009)	(0.009)	(0.009)	(0.009)
3rd Income Quintile * Covid	0.009	0.009	0.009	0.009	0.011	0.010
	(0.009)	(0.009)	(0.009)	(0.009)	(0.009)	(0.009)
4th Income Quintile * Covid	0.030***	0.030***	0.029***	0.029***	0.033***	0.031***
	(0.009)	(0.009)	(0.009)	(0.009)	(0.009)	(0.009)
5th Income Quintile * Covid	0.027***	0.027***	0.026***	0.026***	0.032***	0.031***
	(0.009)	(0.009)	(0.009)	(0.009)	(0.009)	(0.009)
2nd Ethnicity Quintile * Covid	-0.008	-0.008	-0.009	-0.009	-0.007	-0.007
	(0.008)	(0.008)	(0.008)	(0.008)	(0.008)	(0.008)
3rd Ethnicity Quintile * Covid	-0.009	-0.009	-0.009	-0.009	-0.007	-0.007
	(0.008)	(0.008)	(0.008)	(0.008)	(0.008)	(0.008)
4th Ethnicity Quintile * Covid	-0.016*	-0.016*	-0.016*	-0.017*	-0.012	-0.013
	(0.009)	(0.009)	(0.009)	(0.009)	(0.009)	(0.009)
5th Ethnicity Quintile * Covid	-0.065***	-0.065***	-0.065***	-0.066***	-0.058***	-0.059***
	(0.010)	(0.010)	(0.010)	(0.010)	(0.010)	(0.010)
Mean Age * Covid	0.007***	0.007***	0.007***	0.007***	0.008***	0.007***
	(0.002)	(0.002)	(0.002)	(0.002)	(0.002)	(0.002)
% Female * Covid	-0.008***	-0.008***	-0.008***	-0.008***	-0.008***	-0.008***
	(0.002)	(0.002)	(0.002)	(0.002)	(0.002)	(0.002)
Mean Charlson * Covid	0.002	0.002	0.002	0.002	0.001	0.001

	(0.002)	(0.002)	(0.002)	(0.002)	(0.002)	(0.002)
Number of Covid Patients from MSOA (Lagged 0 Weeks)	-76.552***					
	(6.087)					
Number of Covid Patients from MSOA (Lagged 1 Week)		-83.223***				
		(6.346)				
Number of Covid Patients from MSOA (Lagged 2 Weeks)			-71.155***			
			(6.531)			
Number of Covid Patients from MSOA (Lagged 3 Weeks)				-65.088***		
				(6.680)		
Number of Covid Patients from Local Authority (Lagged 1 Weeks)					-391.628***	
					(16.049)	
Number of Covid Patients in Local Hospital (Lagged 1 Weeks)						-441.072***
						(18.646)
Num.Obs.	346332	346332	346332	346332	346332	346332
R2	0.109	0.110	0.109	0.109	0.111	0.111

Source: Authors' calculations using NHS Digital's Hospital Episode Statistics.
Note: Standard errors (clustered at the MSOA level) in parentheses. * p < 0.1, ** p < 0.05, *** p < 0.01. Fixed effects are included.

Results split by diagnosis type

Only primary diagnoses with at least 100 emergency admissions in 2019 are included. We classify primary diagnoses in two ways. First by their in-hospital mortality rates (controlling for patient age year dummy variables and sex), with diagnoses classed as high if their mortality rate is above the 75th percentile. Second by their deferability status, following the methodology of Card, Dobkin and Maestas (2009)[2] with diagnoses classed as non-deferrable if volumes are similar on weekdays and at weekends. There are too few admissions per week per MSOA of each type, so we aggregate to four-week periods. In each case the specification is the same as (6).

Table A10: Outcome variable is non-primary-COVID-19 emergency admissions in 2020 normalized by closest four-week period in 2019.

	Mortality		Deferability	
	Low (6)	High (6)	Deferrable (6)	Non-Deferrable (6)
Intercept	1.031***	1.078***	1.042***	1.043***
	(0.008)	(0.014)	(0.008)	(0.013)
2nd Income Quintile * Covid	0.011	-0.007	0.012	-0.006
	(0.009)	(0.015)	(0.009)	(0.016)
3rd Income Quintile * Covid	0.012	-0.014	0.011	0.011
	(0.009)	(0.016)	(0.009)	(0.015)
4th Income Quintile * Covid	0.040***	-0.008	0.031***	0.024
	(0.009)	(0.015)	(0.009)	(0.015)
5th Income Quintile * Covid	0.038***	-0.025	0.029***	0.020
	(0.009)	(0.015)	(0.009)	(0.015)
2nd Ethnicity Quintile * Covid	-0.014*	0.010	-0.005	-0.026**
	(0.008)	(0.014)	(0.008)	(0.013)
3rd Ethnicity Quintile * Covid	-0.019**	0.028**	-0.010	-0.014
	(0.009)	(0.014)	(0.008)	(0.014)
4th Ethnicity Quintile * Covid	-0.027***	0.009	-0.017*	-0.015
	(0.009)	(0.015)	(0.009)	(0.014)
5th Ethnicity Quintile * Covid	-0.058***	-0.053***	-0.049***	-0.068***
	(0.010)	(0.017)	(0.010)	(0.017)
Mean Age * Covid	0.009***	-0.007*	0.008***	0.007**
	(0.002)	(0.004)	(0.002)	(0.003)
% Female * Covid	-0.007***	-0.006**	-0.008***	-0.004**
	(0.002)	(0.002)	(0.001)	(0.002)
Mean Charlson * Covid	0.001	-0.005**	0.003*	-0.002
	(0.002)	(0.002)	(0.001)	(0.002)
Number of Covid Patients	-32.787***	-46.168***	-29.721***	-48.338***
	(2.819)	(4.894)	(2.748)	(4.487)
Num.Obs.	88283	88276	88283	88281
R2	0.257	0.065	0.253	0.079

Source: Authors' calculations using NHS Digital's Hospital Episode Statistics.
Note: Standard errors (clustered at the MSOA level) in parentheses. * $p < 0.1$, ** $p < 0.05$, *** $p < 0.01$. Fixed effects are included.

Bibliography

1. Office for National Statistics. Estimates of the population for the UK, England and Wales, Scotland and Northern Ireland. (2020).

2. Card, D., Dobkin, C. & Maestas, N. Does Medicare Save Lives? *Q. J. Econ.* **124**, 597–636 (2009).
